# The ability to maintain rhythm is predictive of ADHD diagnosis and profile

**DOI:** 10.1186/s12888-023-05401-8

**Published:** 2023-12-08

**Authors:** Peik Gustafsson, Katarina Kjell, Maurizio Cundari, Martin Larsson, Jenny Edbladh, Guy Madison, Olga Kazakova, Anders Rasmussen

**Affiliations:** 1 Department of Clinical Sciences, Faculty of Medicine, Lund University, Lund, Sweden; 2 Department of Psychology, Faculty of Social Science, Lund University, Lund, Sweden; 3 Department of Experimental Medical Science, Faculty of Medicine, Lund University, Lund, Sweden; 4 Unit of Neuropsychiatry, Hospital of Helsingborg, Helsingborg, Sweden; 5 Unit of Neurology, Hospital of Helsingborg, Helsingborg, Sweden; 6 Modigo, Lund, Sweden; 7 Department of Psychology, Umeå University, Umeå, Sweden

**Keywords:** Attention deficit hyperactivity disorder, Cerebellum, Adolescents

## Abstract

Attention deficit hyperactivity disorder (ADHD) is the most prevalent neuropsychiatric disorder in the world. Currently, the diagnosis is based mainly on interviews, resulting in uncertainties in the clinical assessment. While some neuropsychological tests are used, their specificity and selectivity are low, and more reliable biomarkers are desirable. Previous research indicates that ADHD is associated with morphological changes in the cerebellum, which is essential for motor ability and timing. Here, we compared 29 children diagnosed with ADHD to 96 age-matched controls on prism adaptation, eyeblink conditioning, and timed motor performance in a finger tapping task. Prism adaptation and timing precision in the finger tapping task, but not performance on eyeblink conditioning, differed between the ADHD and control groups, as well as between children with and without Deficits in Attention, Motor control, and Perception (DAMP) – a more severe form of ADHD. The results suggest finger tapping can be used as a cheap, objective, and unbiased biomarker to complement current diagnostic procedures.

## Introduction

Attention Deficit Hyperactive Disorder (ADHD) is one of the most common neuropsychiatric disorders in children and adolescents, with a prevalence of 2.2–7.2% depending on age group, assessment procedures, and location [1–4]. Boys are more likely to be diagnosed with ADHD than girls, with a ratio ranging from 1:3 to 1:16 [5]. A reason for this sex difference might be that ADHD manifests differently in girls, making them less likely to meet the diagnostic criteria [6, 7]. The societal burden of ADHD is high [8]. ADHD has been linked to drug abuse [9], social problems [10], lower occupational and educational achievements [11], higher alcohol intake during pregnancy leading to increased incidence of fetal alcohol spectrum disorders [12], and more criminal activity [13].

The diagnosis of ADHD is most commonly based on the Diagnostic and Statistical Manual of Mental Disorders (DSM-5) issued by the American Psychiatric Association or the International Statistical Classification of Diseases and Related Health Problems (ICD, 11th ed.) [14]. Several lines of research suggest that it is difficult to distinguish ADHD and immaturity, and the youngest children in a specific cohort have a higher risk of being diagnosed with ADHD [15, 16]. Consequently, many are incorrectly diagnosed – which is underlined by the fact that one-third of children diagnosed with ADHD no longer meet the criteria in adulthood [17, 18].

The Deficits in Attention, Motor control, and Perception (DAMP) concept represents symptoms extending those of ADHD with those of Minor Neurological Deficits (MND) [19]. DAMP was introduced in Scandinavia in the 1980s. Around 50 percent of children with ADHD also have MND. These children exhibit stereotypical, repetitive motor behaviors [20] and deficits in gross and fine motor control [21]. Whether ADHD and DAMP should be regarded as separate disorders or if they should be regarded as more or less severe forms of ADHD is an ongoing discussion. There is evidence for shared genetic components [22], while patients with DAMP do have distinct psychological and behavioral profiles [23, 24]. Moreover, individuals with ADHD who also have MND have an even poorer psychosocial prognosis than those affected by only one of these disorders [25].

Many studies have examined brain morphology and functional activation patterns in children with ADHD and MND. The results are heterogeneous, but some systematic patterns have emerged. Analyses of resting-state connectivity of the primary motor cortex revealed that children with MND and ADHD have unique alterations in the functional connectivity between the primary motor cortex and sensory networks compared to children with MND or ADHD only [26]. Historically, ADHD research has focused on the prefrontal cortex and its connections with the striatum [27], but recent research suggests that morphological changes in the cerebellum are a part of the explanation [28–30]. The cerebellum plays a crucial role in motor learning, timing, and coordination of movements [31], but is also involved in working memory, language, cognition, learning, and affective control [32–37].

Several studies have examined links between ADHD and brain morphology, including cerebellar morphology. Multiple studies show that patients with ADHD have a smaller cerebellum [38–45], although there are also some studies questioning this [46–48]. One suggested reason for the heterogeneous results is that sex moderates the association [39, 44, 45], although this has also been questioned [47, 49]. The heterogenous results in previous studies may reflect the fact that ADHD is a heterogeneous disorder and that diagnostic procedures differ significantly. Nevertheless, it is fair to say that the bulk of the evidence suggests that ADHD is linked to cerebellar abnormalities [28], and that the level of cerebellar abnormalities correlates with the severity of symptoms [47].

### Can cerebellar tests be used to support ADHD diagnosis?

If there is a link between ADHD and cerebellar function, tests of cerebellar function could supplement the diagnosis of ADHD, MND, or DAMP. Evidence shows that performance on tasks related to cerebellar function differs between patients and controls. On eyeblink conditioning, children with ADHD exhibit a similar percentage of conditioned responses, but a larger proportion of abnormally timed conditioned responses [50–52]. In terms of motor timing tasks more generally, children and adolescents with ADHD perform worse on and show larger intra-individual variance (IIV) than healthy controls, according to a systematic review [53]. ADHD is also linked with an inability to sense rhythms [54]. When it comes to maintaining a rhythm, the results have been mixed, with one study showing no differences between patients and controls [55] and another study suggesting that ADHD patients exhibit higher tapping variance [56]. In summary, evidence from neuroimaging, clinical, and behavioral studies support the view that ADHD and cerebellar function are closely linked [30], although there are a few exceptions [57, 58].

The present study aims to assess cerebellar function in children with ADHD and DAMP relative to neurotypical developing controls. The test battery consists of five subtests, three of which have been linked to cerebellar function in previous studies: (1) prism adaptation, (2) isochronous serial interval production (ISIP), also known as finger tapping, and (3) eyeblink conditioning. However, individuals who perform well on one of these tasks do not necessarily perform well on the others, suggesting that they rely on partially distinct cerebellar mechanisms [59]. In addition, we administered (4) a neuromotor assessment test to assess motor function and (5) the similarities part of the Wechsler Intelligence Scale for Children (WISC) to assess intelligence. To our knowledge, this is the first study to apply a wide test battery of cerebellar function to children with ADHD and DAMP. Furthermore, we aim to examine the possibility of using one or several of these tests diagnostically. As performance tests, they are less likely to be influenced by subjective bias. They are also cheap and easy to administer and should therefore be easy to introduce in clinical settings. We hypothesize that children with ADHD will exhibit deficits on one or several tests associated with cerebellar function.

## Methods and materials

### Participants

A total of 125 children were tested in this study. The control group consisted of 96 children, 47 boys, and 49 girls, with an average age of 12.0 ± 1.02, recruited from a local elementary school in Lund (Järnåkraskolan). The ADHD group consisted of 29 children diagnosed with ADHD, 20 boys and 9 girls, with an average age of 12.5 ± 2.25. They were recruited from three institutions in southern Sweden: (1) The child and adolescent psychiatry unit in Malmö (BUP), (2) Modigo – a private clinic that assesses and diagnoses children with neurodevelopmental disorders, and (3) Källan Teaching Group – a public teaching group for children and adolescents with special education needs. The children were tested between 2017 and 2020. The study was approved by the Swedish Ethical Review Authority in Lund, Sweden (DNR. 2017/745).

### Test procedure

Upon arrival, the children and their legal guardians received information about the test battery and were given an opportunity to ask questions. The children were informed that they could end their participation at any time without any consequences. One legal guardian per participant was allowed in the test room if the child or the legal guardian so wished. The five tests were performed in the same order for all participants: (1) prism adaptation, (2) ISIP, (3) eyeblink conditioning, (4) a standard neuromotor status test, and (5) the similarities part of the Wechsler Intelligence Scale for Children (WISC). The whole session took about one hour to complete and took place in a quiet room at the school or the clinic, whichever was most convenient for the participant.

### Prism adaptation

Prism adaptation is used to assess the adaptation of motor activity following changes in visual input. Previous research has linked performance on prism adaptation to cerebellar function [60, 61]. In this study, the participant stood at arm's length from a measuring tape attached to the wall. On each trial, the participant was instructed to look at the measuring tape and identify the midpoint of the measuring tape, then close their eyes, and then point to where they believe the target is. While still pointing and standing in the same place, the participant opened their eyes to see where they had pointed. This procedure is repeated 10 times. For the next 10 trials, the participants wore the prism glasses that displaced the visual field laterally by 15 degrees. Wearing the prisms usually results in a pointing error that gradually decreases as the participant adapts to the new visuomotor relationship. In the last ten trials (21–30), the glasses were taken off again. Removing the glasses typically results in a pointing error in the direction opposite to the error that occurred when putting the prisms on – often to the surprise of the participant. On each trial, the experimenter then wrote down the error in centimeters. A positive value indicates that the participant pointed to the left of the target, and a negative value indicates that the participant pointed to the right of the target. For the statistical analysis we analyzed the error on the first trial after putting the glasses on and the first trial after taking the glasses off (trials 11 and 21).

### Finger tapping

Two types of finger tapping performance were assessed, formally known as sensorimotor synchronization (SMS) and isochronous serial interval production (ISIP), typically combined in the so-called synchronization-continuation paradigm [62], known to activate the cerebellum [63]. In this task, the participant listens to a rhythmic sound sequence and is asked to synchronize with the sounds by pressing the space bar on a laptop with the dominant hand (the SMS part), and to continue pressing the space bar at the same pace when the rhythmic sound stopped (the ISIP part) until they hear a stop signal. This procedure was repeated five times. Each repetition included 15 paced intervals followed by 70 self-paced intervals. The inter-onset interval (IOI) was set to 524 ms because this tends to be in the middle of an optimal range where the task feels easy, and the variability is the smallest [64–66].

The SMS and the ISIP data were analyzed separately. Two metrics were used for the SMS data: the mean inter-tap interval and the mean asynchronies relative to the guiding sounds. Three metrics were used for the ISIP data: the mean inter-tap interval and two measures of variability called drift and local [64]. These measures were used because there is a tendency to change and typically increase the frequency of the button presses over time in the ISIP phase (a phenomenon known as drift), making the difference between the initial target interval and the current production interval less relevant. Instead, our metrics capture both the magnitude of the drift in the rhythm that the participant actually produces, and the local variability in the participant’s ability to maintain that rhythm. Response IOIs shorter than 400 ms and longer than 650 ms were excluded from the analyses. Such extreme intervals were uncommon and typically depended on a button press not being registered or, in some cases, a lack of concentration on behalf of the participant.

### Eyeblink conditioning

Eyeblink conditioning is one of the most used tests when studying cerebellar function. Many studies corroborate its cerebellar dependence. In this task, a conditional stimulus (CS) is paired with an unconditional stimulus (US) that provokes an unconditional response (UR). Over time, the CS will produce a conditional response (CR), even when the US is absent [67–70]. Participants were equipped with headphones mounted with a flexible plastic tube for administering an air puff to the eye and a magnetic sensor. To register eyelid movements we attached a 0.5 g neodymium magnet to the participant's left eyelid. As a result, eyelid movements caused a change in the magnetic field that the magnetic sensor picked up. During the test, the children watched a cartoon (Shaun the Sheep) and were asked to focus on the film and not pay attention to the stimuli or their reactions to it.

The CS consisted of sinusoidal 1,000 Hz auditory tone with a duration of 1 s. Before the training, we ensured the participant could hear the tone clearly. The US was a ~ 100-ms air puff to the cornea with a pressure between 0,5–1,5 bar. The exact pressure was individually adjusted so that the air puff triggered an eyeblink reflex (a UR) while not being perceived as overly unpleasant. Before starting the tests, we made sure that the participant could hear the tone and that the tone itself did not trigger a blink reflex. We used two different interstimulus intervals (the time between the onset of the CS and US) of 300 and 500 ms. We used 500 ms because that is a common interstimulus interval in human studies. We also added a shorter 300 ms interstimulus interval because it is closer to what is typically used in animal studies and because we reasoned that it would reduce the probability of volitional interference [71].

The inter-trial interval (ITI) was set to vary randomly from 4 to 12 s. Training consisted of 100 trials with 20% randomly interspersed probe trials where the CS was presented without the US. Measurements from the magnetic sensor were sampled using a Micro 1401 AD converter (Cambridge Electronics Design) connected to a PC running Spike2 v.9 (Cambridge Electronics Design). The raw signal from the magnetic sensor was exported to Matlab, where custom scripts were used to identify the timing of blink responses relative to the CS onset. Trials were categorized as invalid if an eyelid movement occurred within 100 ms of the CS onset. On valid trials, the script determined if there was a blink response and then, based on the onset of the response, whether that response should be categorized as a CR. A CR was defined as a blink response with an onset before the air puff. For each participant, we visually inspected 10 randomly selected trials to ensure that the algorithm worked for most trials. While a few categorizations errors did occur, we did not correct them so as not to introduce any subjective bias into the evaluation.

### Neuromotor test

The fourth test in the battery was a standardized neuromotor test consisting of several short motor tasks [72], including a diadochokinesia test (quick alternating pronation and supination movements), Precthls test [73], standing on one leg, jumping on one leg, walking on the heels, and Bishop’s test (using both hands to draw two squares simultaneously). The experimenter scored performance on each task on a 0–2 scale. A zero meant the child did not exhibit any difficulties. A score of 2 meant severe difficulties. The total score (0–26) on the neuromotor test was used to distinguish between children with ADHD and children with DAMP, where MND was defined as having a score of 4 or higher. Participants with ADHD and MND were categorized as having DAMP.

### Intelligence test

To assess and control for IQ we administered the similarities part of WISC. In this test children should state in what way to concepts are similar. For example, if the experimenter says apples and bananas are both …, then the child should say fruits or provide and explanation that is equivalent of this statement. The performance on this test was used as a proxy for IQ.

### Statistical analysis

Since several variables were not normally distributed, non-parametrical statistical tests were used to test statistical significance. The Mann–Whitney U-test was used for comparisons of the ADHD group and the control group, and the Kruskal–Wallis test for comparisons between children with ADHD without MND, children with DAMP (defined as having both ADHD and MND), children with MND without ADHD, and children without ADHD and MND, with the Mann–Whitney U-test for post hoc analyses. A logistic regression analysis was conducted to assess the various tests’ predictive value for detecting DAMP. Spearman correlations were performed to analyze the associations between the different test variables. ROC analyses were performed to investigate the sensitivity and specificity of the tests in predicting DAMP. Cut-off values with the highest simultaneous sensitivity and specificity were made, and the sensitivities and specificities were calculated. Exact p-values are reported in the table.

## Results

### Participants

First, we consider differences between the ADHD and control groups. Table 1 provides an overview of the key variables and their distributions. The ADHD group had a higher percentage of boys compared to the control group – reflecting that the diagnosis is more common in boys than girls. Likewise, MND (> 4 points on the neuromotor test) was more common in the ADHD group. Since the ratio of boys and girls differed between the control and experimental groups, we used independent t-tests to test if any of our performance measures differed between boys and girls. This was not the case (all *p* > 0.18). Since there was no sex difference in this sample, we did not consider sex further in the remaining analysis.

**Table 1:** Descriptive statistics for the two groups, including median, interquartile range (IQR), and the number of participants for key measures included in the study. Differences between the study and control groups are assessed with the Mann–Whitney U test

**Variable**	**ADHD**	**Controls**	***p***
Age (years)	12.68 (10.95–14.12), *n* = 29	11.75 (11.22–12.91), *n* = 96	0.11
Sex (male/female)	20 males; 9 females	47 males; 49 females	0.043*
NM scores (ms)	2.0 (0.0–5.0), *n* = 27	0.00 (0.00–1.00), *n* = 96	< 0.001***
Eyeblink onset 300 (ms)	242.9 (212.6–303.4), *n* = 9	241.3 (215.9–292.0), *n* = 55	0.451
Eyeblink onset 500 (ms)	345.6 (324.5–387.8), *n* = 17	359.0 (337.2–402.6), *n* = 38	0.099
Onset SD 300 (ms)	87.1 (69.2–123.1), *n* = 9	93.5 (80.3–119.7), *n* = 55	0.395
Onset SD 500 (ms)	107.3 (92.1–137.7), *n* = 17	119.7 (108.3–142.4), *n* = 38	0.064
Percent CR 300 (%)	27.1 (20.1–41.0) *n* = 9	18.3 (8.6–37.6) *n* = 55	0.428
Percent CR 500 (%)	46.9 (30.9–78.9), *n* = 17	46.5 (33.4–72.8), *n* = 38	0.518
Prism first with (cm)	10.00 (2.25–13.75), *n* = 28	14.00 (10.00–20.00), *n* = 96	< 0.001***
Prism first without (cm)	-4.50 (-7.0–0.0), *n* = 28	-7.00 (-10.00- -2.50), *n* = 96	0.025*
Sync Mean (ms)	516.7 (507.5–521.2), *n* = 29	522.6 (518.4–525.6), *n* = 95	< 0.001**
Sync SD (ms)	36.9 (31.5–45.8), *n* = 29	33.2 (27.1–37.6), *n* = 95	0.009**
Production mean (ms)	488.2 (469.6–503.2), *n* = 29	500.7 (487.8–520.7), *n* = 95	0.003**
Local (ms)	32.84 (27.50–50.19), *n* = 29	29.90 (25.18–36.56), *n* = 95	0.033*
Drift (ms)	13.49 (9.72–33.16), *n* = 29	10.58 (7.59–16.18), *n* = 95	0.009**

The asterisks show the significance level (* = *p* < 0.05; ** = *p* < 0.01; *** = *p* < 0.001)

### Cerebellar tests

#### Prism adaptation

**Fig. 1:**
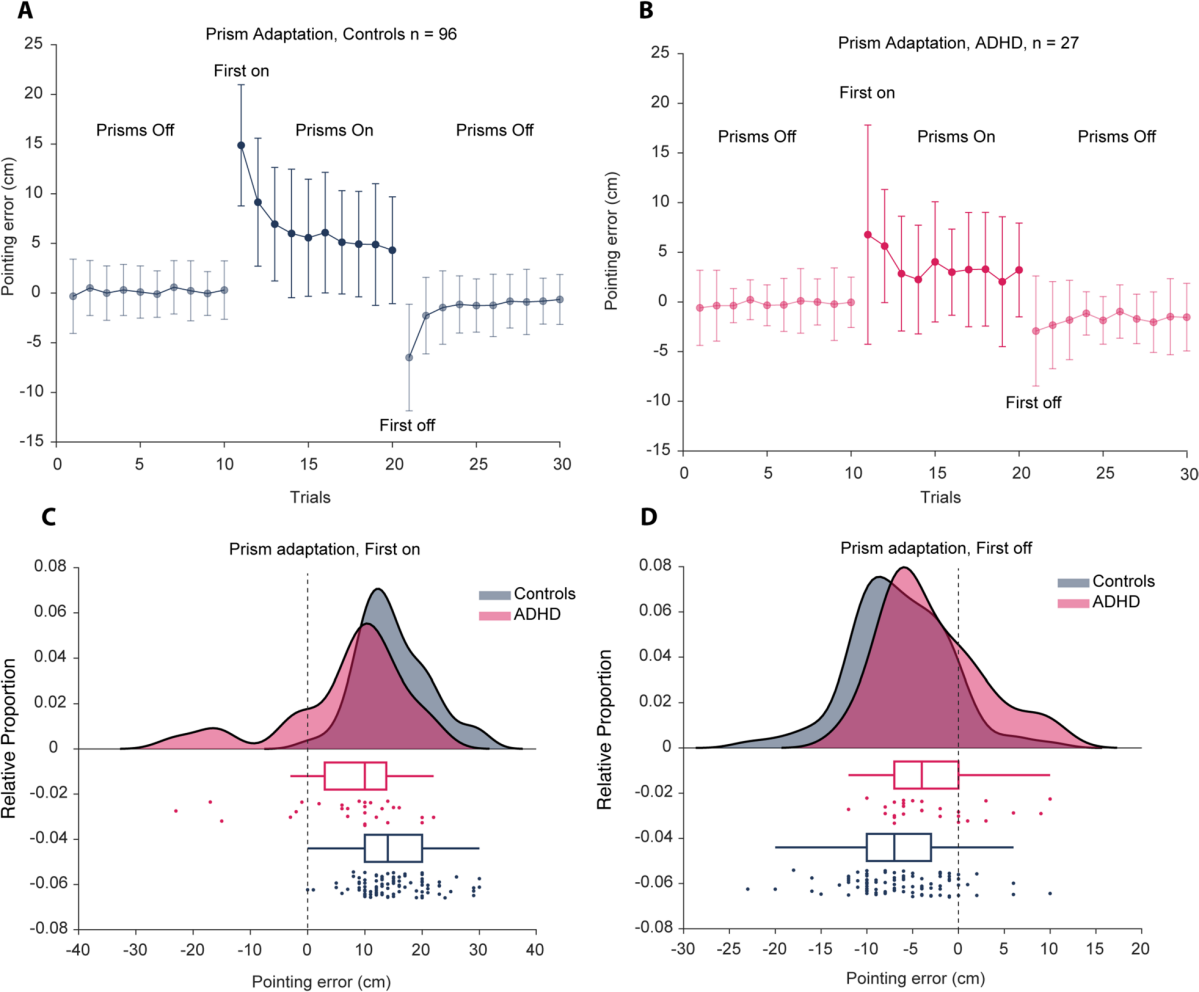
Prism adaptation. **A**, **B** shows mean ± standard deviation of the pointing errors on prism adaptation. The raincloud plots below (**C**, **D**) show the mean and the spread of pointing errors on the first trial with the prisms on and the first trial without the prisms

To assess whether children with ADHD differed from controls on prism adaptation, we examined two variables, the error on the first trial after putting the prisms on (prism first with) and the first trial after taking the prisms off (prism first without). As illustrated in Fig. 1, the level of adaptation differed between the ADHD group and the controls. Children with ADHD produced smaller errors after putting the glasses on and smaller errors after taking the glasses off (see Table 1 and Fig. 1). Note that smaller errors do not mean better performance since the adaptive strategy is to change motor programs in response to an altered visuomotor relation.

#### Eyeblink conditioning

**Fig. 2:**
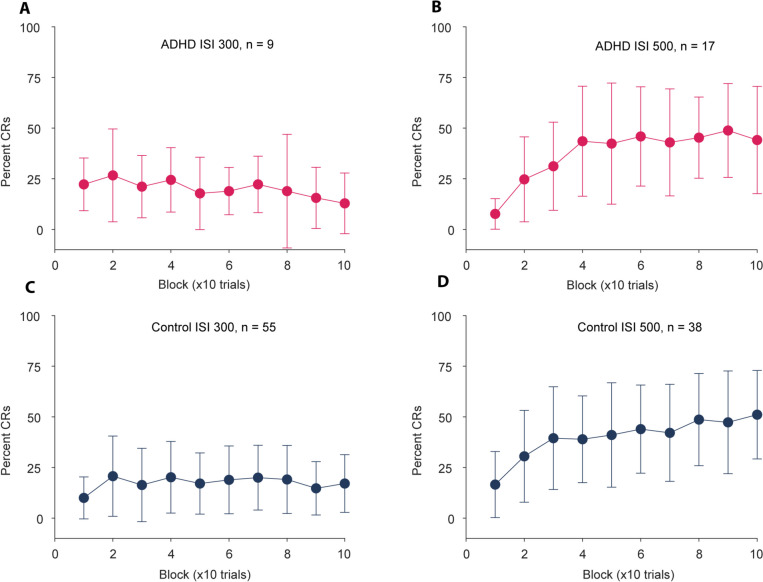
Percent CR on eyeblink conditioning. The different panels show the percentage of conditioned responses on successive blocks of 10 trials during training in an eyeblink conditioning paradigm. Participants were trained either with an interstimulus interval of 300 ms (**A** & **C**) or 500 ms (**B** & **D**). The performance of participants with ADHD is shown in A and B, and panels C and D show the performance of control participants

**Fig. 3:**
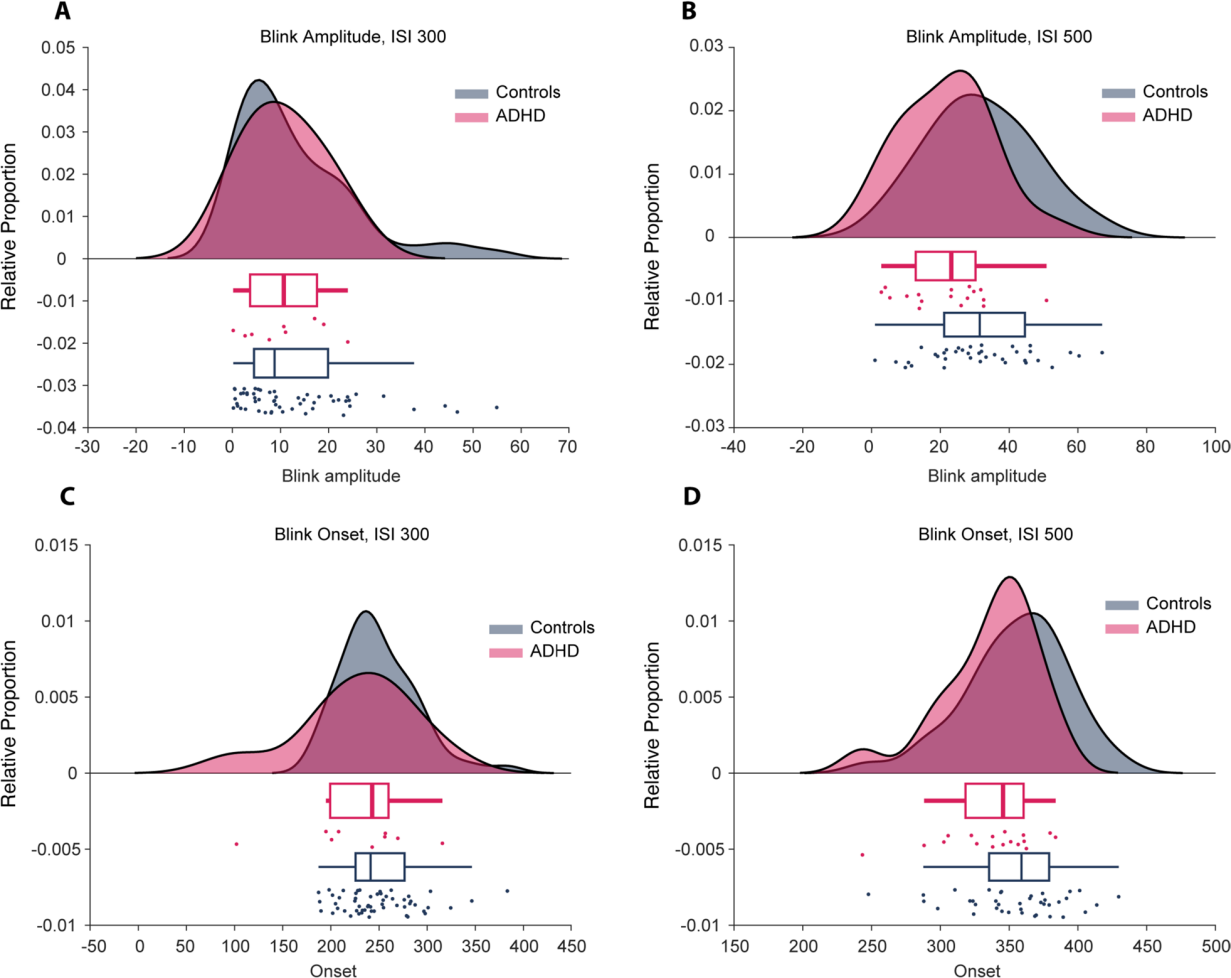
Raincloud plots [74] illustrating the blink amplitude and blink onset of conditioned blink responses. Panels **A**, **B** shows the amplitude of conditioned blink responses for children with ADHD and controls, conditioned with either a 300 ms or 500 ms ISI. Panels **C**, **D** shows the blink onset. There were no statistically significant differences in any of these variables

No differences or trends were found for any of the variables examined for eyeblink conditioning. We compared the percentage of conditioned responses (Percent CR), the onset of conditioned responses (Eyeblink onset), and the variation in the onset of conditioned responses (Eyeblink onset SD) (all *p*-values > 0.3). As illustrated in Figs. 2 and  3, conditioning was poor in both groups, especially when using a 300 ms ISI. The maximum percentage of conditioned responses on a block of 10 trials barely exceeded 50% after 100 trials. Perhaps a longer training regimen would have yielded a higher percentage of conditioned responses, thus providing a better test of whether children with ADHD differ from controls on eyeblink conditioning.

#### Finger tapping

**Fig. 4:**
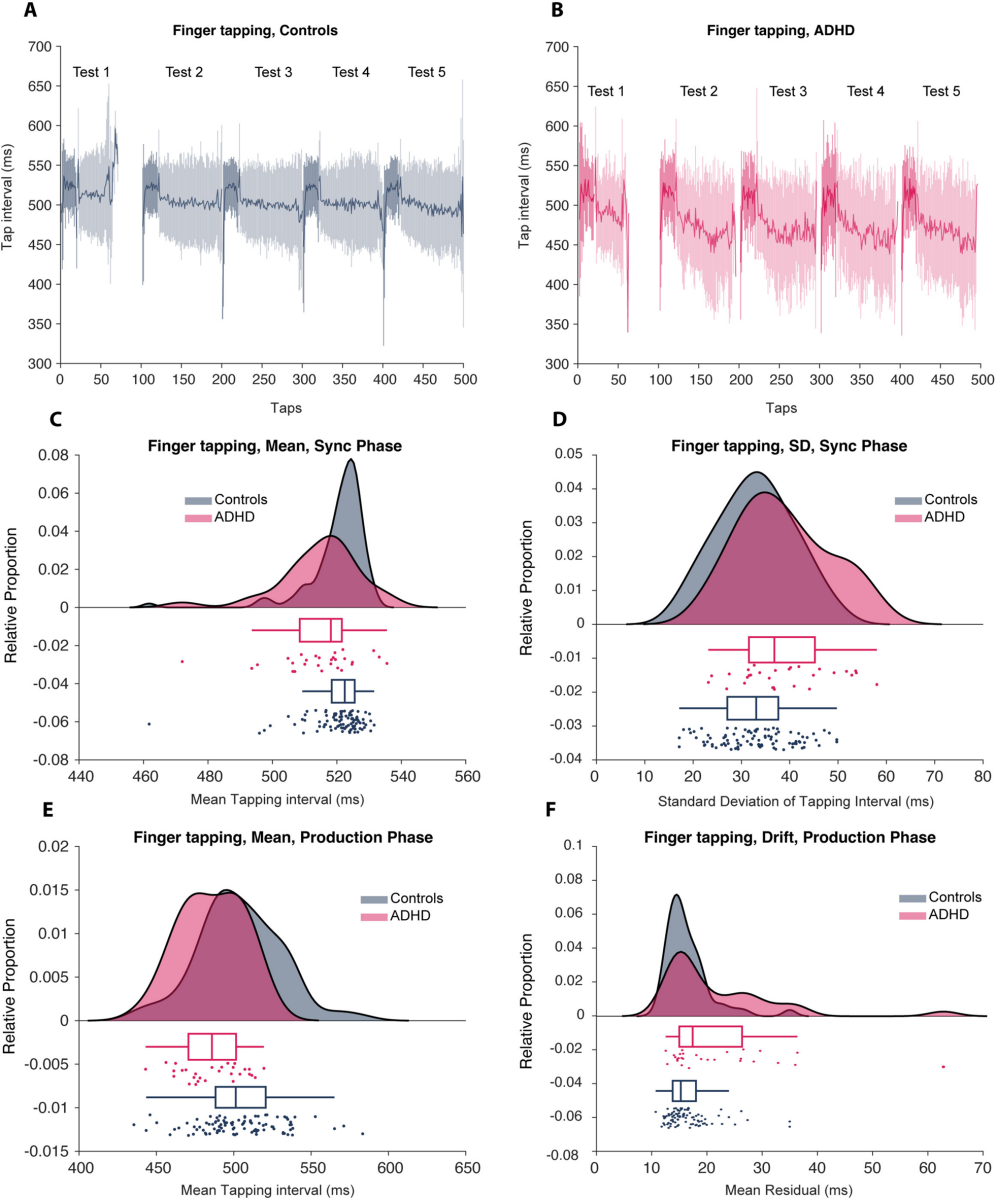
Finger tapping. **A**-**B** shows mean ± standard deviation of the IOIs on five successive sessions. The initial, darker parts of each test represent the SMS phase, with the remaining trials representing the ISIP phase (without the sound). The raincloud plots below (**C**-**F**) show the mean and the variance of IOIs intervals for the SMS phase (**C**-**D**) and the ISIP phase (**E**–**F**)

Each of the five trials began with the participant attempting to synchronize responses to an auditory rhythm (SMS), which was seamlessly followed by a production phase (ISIP) when the sounds stopped. As mentioned above, the metrics used for SMS were mean inter onset interval (IOI) and asynchronies and for ISIP mean (IOI), local and drift variability. Comparing the ADHD and controls revealed significant differences in IOI and variability in both the SMS and ISIP phases (see Table 1). Participants with ADHD had a shorter mean IOI and also exhibited more significant variability during both phases (see Fig. 4).

#### DAMP

Next, we examined whether performance on our test battery could be used to identify participants with DAMP. The ADHD diagnosis and the control children were divided into subgroups according to the presence or absence of MND. The four resulting groups were then compared with respect to the prism adaptation and finger-tapping tests (see Table 2). Eyeblink conditioning was not examined further because there were no main effects for any of the variables tested. DAMP was defined as a diagnosis of both ADHD and MND according to the neuro-motor test. Post hoc comparisons were done with pair-wise Mann–Whitney U-tests.

**Table 2:** Comparisons between the test variables for the different groups of children (Children with ADHD without MND, children with DAMP, children with MND without ADHD, and children without ADHD and MND). Statistical significance was assessed with the Kruskal–Wallis test

	**ADHD -MND,** ***n* = 18** **12 Boys, 6 Girls**	**ADHD + MND,** ***n* = 10** **7 Boys, 3 Girls**	**Controls + MND,** ***n* = 13** **6 Boys, 7 Girls**	**Controls -MND,** ***n* = 84** **42 Boys, 42 Girls**	***p***
Age	12.9 (11.6–14.1)	11.3 (9.5–14.2)	11.6 (11.3–11.8)	11.8 (11.2–13.1)	0.058
NM scores	1.00 (0.00–2.00)	5.00 (4.75–6.75)	5.00 (4.50–6.00)	0.00 (0.00–0.00)	< 0.001
Prism first with	10.0 (5.00–14.5)	6.5 (-5.25–12.3)	12.0 (10.0–14.0)	14.0 (10.3–20.0)	< 0.001
Prism without	-5.50 (-7.00–0.0)	-3.00 (-7.0–0.25)	-7.0 (-10.0- -4.5)	-6.50 (-10.0- -2.0)	0.163
Sync mean	519 (513–522)	510 (502–519)	522 (512–525)	523 (519–526)	0.002
Sync SD	32.3 (29.9–40.0)	45.1 (39.9–53.7)	33.7 (32.7–36.9)	32.7 (26.7–38.7)	< 0.001
Prod mean	499 (475–510)	473 (461–491)	489 (440–503)	506 (489–524)	0.001
Local	29.9 (26.1–35.2)	53.8 (37.7–62.5)	31.3 (27.9–34.2)	29.9 (25.0–37.0)	0.002
Drift	11.0 (9.1–17.2)	36.9 (29.7–49.2)	12.3 (9.9–15.8)	10.1 (7.5–16.2)	< 0.001
WISC	23 (19–29)	22 (17–23)	18 (16–18)	20 (16–23)	0.148

Significant differences were found between the children with ADHD without MND and the children with DAMP concerning the variables Sync SD (*p* < 0.001), Prod mean (*p* = 0.024), Local (*p* < 0.001), and Drift (0.001). Significant differences were found between the children with DAMP and control children with MND concerning Sync sd (*p* < 0.001), Prod mean (*p* = 0.049), Local (*p* = 0.002), and Drift (*p* = 0.001). Significant differences were found between the children with DAMP and the control children without MND, concerning Prism first with (*p* < 0.001), Sync mean (*p* = 0.001), Sync sd (*p* < 0.001), Prod mean (*p* < 0.001), Local (*p* < 0.001), and Drift (*p* < 0.001). Finally, significant differences between the children with ADHD without MND and the controls without MND were found on variables Prism first without (*p* = 0.006) and Sync mean (*p* = 0.010). No significant differences were found between the children with ADHD without MND and the controls with MND, or between the control children with MND and the control children without MND. The sensitivity and specificity of the different variables concerning DAMP were assessed with Receiver Operating Characteristic (ROC) analyses.

#### ROC analysis

**Fig. 5:**
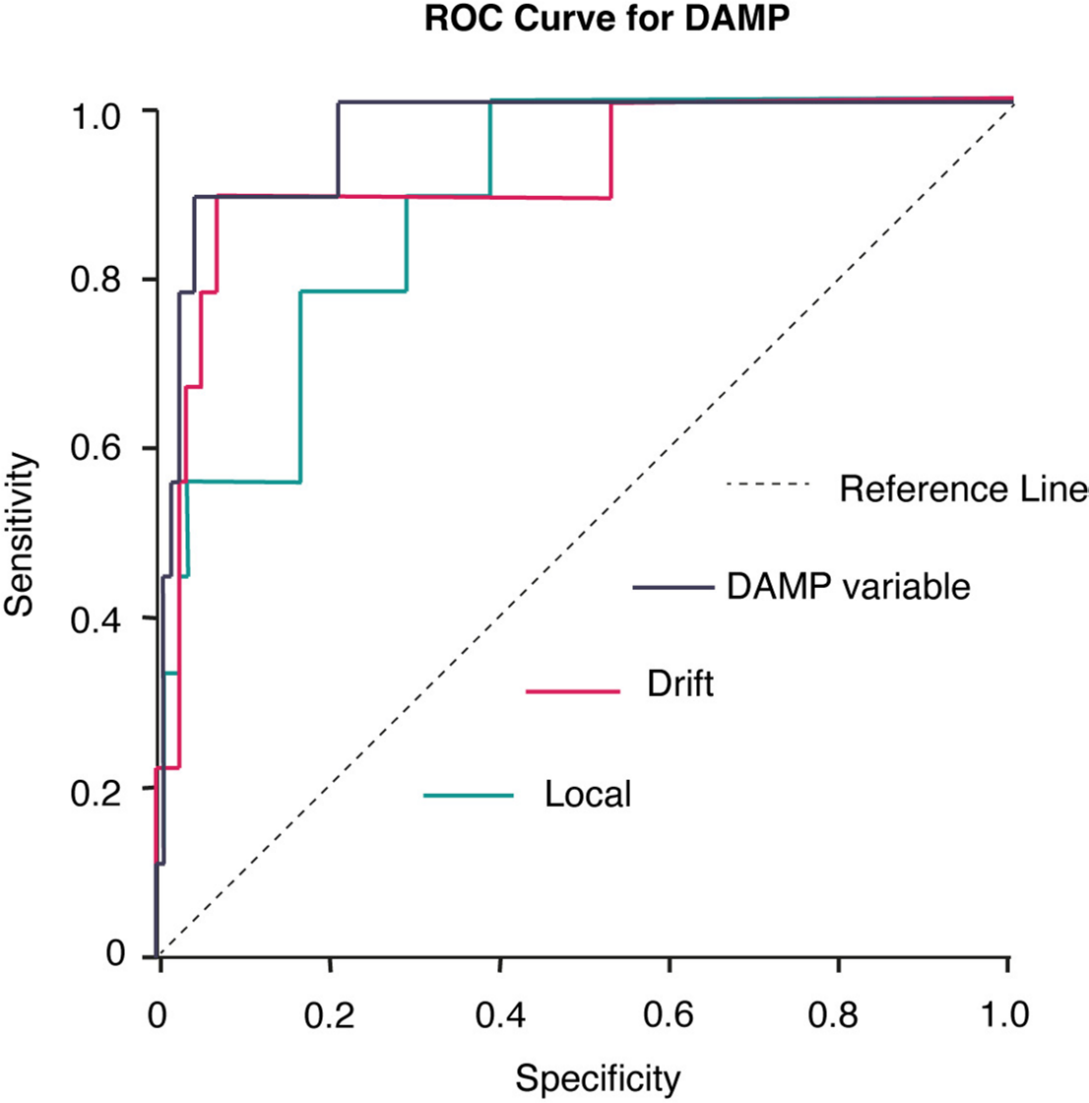
ROC Curves. ROC curves and area under the curve values for key variables in the study. The graph displays the specificity and sensitivity in predicting DAMP among all the children in the study. Since several variables showed promising sensitivity and specificity, we created a new combined DAMP variable This derived variable yielded an AUC of 0.961. With a Cut-off (giving the best simultaneous sensitivity and specificity according to the ROC-curve): >  = -879.4687 defined as 1, else 0 creating a dichotomized DAMP-prediction variable gave a sensitivity of 0.89 and a specificity of 0.96 in predicting DAMP

ROC curves and area under the curve (AUC) values for the variables with high values concerning AUC (above 0.80) can be found in Fig. 5. Since several variables showed good sensitivity and specificity, we created a new combined DAMP variable which was defined as: Sync SD + Local + Drift –Prism first with – Sync Mean – Prod Mean. This derived variable yielded an AUC of 0.961. With a Cut-off (giving the best simultaneous sensitivity and specificity according to the ROC-curve): >  = -879.4687 defined as 1, else 0 creating a dichotomized DAMP-prediction variable showed a sensitivity of 0.89 and a specificity of 0.96 in predicting DAMP. Only dichotomizing variable Drift gave a sensitivity of 0.89 and a specificity of 0.93.

When only children with ADHD-diagnoses were analyzed with the variable Drift dichotomized at the cut-off value giving the best simultaneous sensitivity and specificity values in predicting DAMP, both the sensitivity and specificity were found to be 0.89. When we analyzed the odds for DAMP using univariable logistic regression (Table 3), we found that all finger-tapping variables and the variable Prism First caused an increase in the odds of having DAMP. We omitted the variable Local from the analysis since a strong correlation was found between the variables Local and Drift. When analyzed with multivariable logistic regression, only prism first with and Drift remained significant, pointing to these variables as especially important for predicting DAMP. Controlling for sex did not change these results. These variables were shown to be highly associated with each other.

**Table 3:** Results from logistic regression analyses (univariable and multivariable) with dependent variable DAMP (ADHD + MND), *n* = 124, R^2^ for the multivariable analysis = 0.694

	**Odds ratio** **Univariable logistic regression**	**95% confidence interval**	**Odds ratio Multivariable logistic regression**	**95% confidence interval**
Prism first with	0.906**	0.852–0.963	0.866*	0.763–0.984
Sync mean	0.947*	0.904–0.991	0.966	0.880–1.061
Sync SD	1.250***	1.114–1.403	1.104	0.904–1.348
Prod mean	0.956**	0.928–0.986	0.963	0.897–1.034
Drift	1.127***	1.063–1.195	1.128*	1.020–1.247

The asterisks show the significance level (* = *p* < 0.05; ** = *p* < 0.01; *** = *p* < 0.001)

## Discussion

Our results show that finger tapping and prism adaptation performance can differentiate between children with and without ADHD and even more accurately between children with and without DAMP. As both types of tests are related to cerebellar function, the results reinforce the view that children with ADHD and DAMP have altered cerebellar function leading to deficits in tasks relying on cerebellar function. The high sensitivity and specificity found for these tests point towards their possible clinical utility, especially the finger tapping test, which is easy and quick to administer. Previous research shows that children with DAMP have more learning difficulties and more autistic traits than children with ADHD without MND [75]. Thus, SMS and ISIP performance might be used for identifying children with DAMP, which is of interest since they have a distinct clinical profile accompanied by other risks than children with ADHD but without DAMP. The variation in the ability to maintain a self-paced rhythm (ISIP) has excellent sensitivity and specificity, particularly for the Drift component. This variable alone seems to be an effective biomarker for ADHD and DAMP. Since there were no significant differences between the groups on the WISC subtest, the observed differences in prism adaptation and finger tapping are unlikely to be dependent on intellectual function. Thus, finger tapping – a simple task that takes less than 10 min to perform – can potentially be used in clinical settings to improve evaluations by making them faster and more objective, saving time and money.

ROC analyses showed high values for AUC for several cerebellar-related performance variables, especially measures of finger-tapping variation. When it came to finding children with DAMP, the variable Drift had an especially good relation between sensitivity and specificity – higher than quantitative tests currently used for ADHD evaluationss, such as the Integrated visual and auditory continuous performance test (IVA) [76]. Our derived variable that combined performance on finger tapping and prism adaptation gave an even higher AUC value of 0.961, indicating excellent specificity and sensitivity. Then again, in a clinical setting, the relatively small benefit of adding an extra task should be weighed against the extra time needed to complete that task.

Performance on finger tapping and prism adaptation have been linked to cerebellar performance. The fact that performance on both tests correlated with ADHD diagnosis and DAMP reinforces the view that ADHD is linked to abnormal cerebellar function. We were, therefore, surprised to find that performance on eyeblink conditioning, another test linked to cerebellar function, showed no significant association with ADHD. This appears to contradict Frings et. al., [50], who reported that the timing of conditioned responses is inferior in children with ADHD. However, Frings et. al., aimed the airpuff at the skin close to the eye rather than directly on the cornea and they also used a shorter training protocol – 50 trials instead of 100. Whether these differences in methodology explains the discrepancy in the results is difficult to tell. Another explanation is that there was no effect because conditioning was poor overall. Consistent with our previous study [77], children trained with a 300 ms ISI rarely reached a CR percentage above 30%, and many children barely produced any conditioned responses. Perhaps more trials over multiple days would yield better conditioning and, consequently, a better opportunity to determine whether children with ADHD and DAMP differ from controls on eyeblink conditioning. Based on our results, eyeblink conditioning does not seem to be viable in a clinical setting, given that it takes at least ~ 30 min to complete and does not appear to distinguish the different clinical groups. Nevertheless, given that eyeblink conditioning is the task that is most clearly linked to the cerebellum, it makes sense to keep exploring different protocols, perhaps with new technologies.

Overall, our results support the hypothesis that children with ADHD and DAMP have altered cerebellar functions and that cerebellar performance tests can be helpful in a clinical context. Compared to other potential biomarkers, such as MRI scans, these tests are fast and easy to conduct, making them a more viable option in most settings. The cerebellum has also been linked to autism [78], and future studies should investigate whether these patients display a similar pattern of deficits in cerebellar tasks. While promising, this study also has some limitations. One limitation is that our analysis did not take treatment into account. For children with ADHD, treatment includes psychotherapy and medication, both of which could affect the outcomes of our tests. Moreover, though our overall sample size was large (125 children), it included only 29 children with ADHD. Although the statistical tests revealed several highly significant effects, the results should be independently validated on new datasets. Also, we cannot take for granted that the same pattern is present in adults since we only included children.

Taken together, our results suggest that cerebellar tests, generally and finger tapping in particular, may prove to be a robust research and diagnostic tool in the evaluation of children suspected of having ADHD. Finger tapping seems especially useful when distinguishing between children with ADHD of different severity.

## Data Availability

The datasets and code used will be made available from the corresponding author on reasonable request. Data and code will be made available on a github repository upon acceptance of the article.
